# Complementary surveillance strategies are needed to better characterise the epidemiology, care pathways and treatment outcomes of tuberculosis in children

**DOI:** 10.1186/s12889-018-5252-9

**Published:** 2018-03-23

**Authors:** Karen du Preez, H. Simon Schaaf, Rory Dunbar, Elisabetta Walters, Alvera Swartz, Regan Solomons, Anneke C. Hesseling

**Affiliations:** 1 Desmond Tutu TB Centre, Department of Paediatrics and Child Health, Faculty of Medicine and Health Sciences, Stellenbosch University, Cape Town, South Africa; 2 Tygerberg Hospital, Department of Paediatrics and Child Health, Faculty of Medicine and Health Sciences, Stellenbosch University, Cape Town, South Africa; 3 Western Cape Department of Health, Cape Town, South Africa

**Keywords:** Tuberculosis, Children, Surveillance, Hospital, Burden, Disease spectrum, Care pathways, Outcomes, HIV

## Abstract

**Background:**

Tuberculosis (TB) in young and HIV-infected children is frequently diagnosed at hospital level. In settings where general hospitals do not function as TB reporting units, the burden and severity of childhood TB may not be accurately reflected in routine TB surveillance data. Given the paucibacillary nature of childhood TB, microbiological surveillance alone will miss the majority of hospital-managed children. The study objective was to combine complementary hospital-based surveillance strategies to accurately report the burden, spectrum and outcomes of childhood TB managed at referral hospital-level in a high TB burden setting.

**Methods:**

We conducted a prospective cohort study including all children (< 13 years) managed for TB at a large referral hospital in Cape Town, South Africa during 2012. Children were identified through newly implemented clinical surveillance in addition to existing laboratory surveillance. Data were collected from clinical patient records, the National Health Laboratory Service database, and provincial electronic TB registers. Descriptive statistics were used to report overall TB disease burden, spectrum, care pathways and treatment outcomes. Univariate analysis compared characteristics between children identified through the two hospital-based surveillance strategies to characterise the group of children missed by existing laboratory surveillance.

**Results:**

During 2012, 395 children (180 [45.6%] < 2 years) were managed for TB. Clinical surveillance identified 237 (60%) children in addition to laboratory surveillance. Ninety (24.3%) children were HIV co-infected; 113 (29.5%) had weight-for-age z-scores <− 3. Extra-pulmonary TB (EPTB) was diagnosed in 188 (47.6%); 77 (19.5%) with disseminated TB. Favourable TB treatment outcomes were reported in 300/344 (87.2%) children with drug-susceptible and 50/51 (98.0%) children with drug-resistant TB. Older children (OR 1.7; 95% CI 1.0–2.8), children with EPTB (OR 2.3; 95% CI 1.5–3.6) and in-hospital deaths (OR 5.4; 95% CI 1.1–26.9) were more frequently detected by laboratory surveillance. TB/HIV co-infected children were less likely to be identified through laboratory surveillance (OR 0.3; 95% CI 0.2–0.5).

**Conclusions:**

The burden and spectrum of childhood TB disease managed at referral hospital level in high burden settings is substantial. Hospital-based surveillance in addition to routine TB surveillance is essential to provide a complete picture of the burden, spectrum and impact of childhood TB in settings where hospitals are not TB reporting units.

## Background

In 2016, South Africa remained the country with the highest estimated total tuberculosis (TB) incidence globally (781 per 100,000 population) with an estimated total of 438,000 TB cases, 58,000 occurring in children < 15 years [1]. The South African National TB Programme (NTP) has a decentralized model of TB care, supporting adult and paediatric patients to primarily receive diagnostic and treatment services at community-based primary healthcare (PHC) facilities [2]. Routine TB recording and reporting tools, including TB treatment registers, are therefore typically located at community-based PHC facilities. Data from the TB registers are captured and aggregated into two electronic registers used routinely for reporting NTP TB surveillance data: ETR.Net (drug-susceptible TB [DS-TB]), and EDRWeb (drug-resistant TB [DR-TB]). In the Western Cape Province, general hospitals do not function as TB reporting units therefore inclusion of adults and children in routine TB surveillance data rely on patients accessing treatment at PHC facilities.

In the absence of TB preventive therapy, young children (< 5 years of age) have a high risk of progressing to TB disease and to severe forms of TB once infected with *Mycobacterium tuberculosis (M. tb)* [3]. Young children are also frequently diagnosed with TB at the referral healthcare level (secondary or tertiary hospital) due to diagnostic challenges, including the ability to obtain adequate samples for TB microbiological testing. Children may frequently move between community and hospital-based healthcare services during the course of TB diagnosis and treatment [4]. A previous audit at a large referral hospital in this setting, Tygerberg Hospital (TBH) found that only 62% of children with culture-confirmed TB were included in routine TB surveillance data during 2007–2009, and that children with TB meningitis and children who died in hospital were more likely not to be included in NTP surveillance data [5]. In settings where hospitals do not function as TB reporting units, hospital-based surveillance data is critical to supplement NTP surveillance data in order to have an accurate reflection of the burden and spectrum of TB in children.

Hospital-based laboratory surveillance of childhood TB has provided valuable insight into the epidemiology and trends in drug resistance in the Western Cape Province since 2003 [6–10]. However, given the paucibacillary nature of TB in children with only 25–40% of children treated for TB expected to have bacteriologically confirmed disease [11–13], laboratory surveillance in isolation will miss the majority of children with TB managed at hospital-level. Hospital-based clinical surveillance strategies are therefore needed to identify children with an unconfirmed, clinical TB diagnosis.

In order to document the true burden and spectrum of childhood TB managed at a large referral hospital in a TB endemic setting we conducted a prospective one-year, hospital-based surveillance study at TBH, Cape Town. We implemented new clinical surveillance activities in addition to existing laboratory surveillance to identify all children routinely diagnosed with or treated for TB. We compared TB disease spectrum, clinical characteristics, care pathways and treatment outcomes between children identified through the two complementary hospital-based surveillance strategies, to characterise the group of children that would otherwise be missed by existing laboratory surveillance.

## Methods

### Setting

The Western Cape Province reported the third highest TB incidence rate (681 per 100,000 population) of the nine provinces in South Africa in 2015 [14]. During 2013, 34,880 newly diagnosed patients including 5,919 (17.0%) children < 15 years of age, with DS-TB were reported in routine provincial TB surveillance data [15]. Prevalence of HIV co-infection increased with age, from 5% amongst children < 5 years of age and reaching a high of 46% amongst adults ≥25 years [15].

TBH is a tertiary referral hospital in the Western Cape Province and serves approximately 50% of the paediatric population in the province. TBH has 10 paediatric wards with 268 general and neonatal beds, and had 15,133 admissions with an overall bed occupancy of 80% during 2012 [16]. TBH serves as referral hospital for uncomplicated and complicated TB cases from surrounding high-burden communities and complicated TB cases from more remote areas, and provides secondary level paediatric care to children living in adjacent communities. Children who are medically stable, but require prolonged hospitalisation for medical or social reasons, are referred to dedicated TB hospitals/care facilities.

Children with pulmonary DS-TB are routinely treated for 6 months with a standard first-line drug regimen, consisting of isoniazid, rifampicin and pyrazinamide with or without ethambutol, depending on disease severity [2]. Children with osteoarticular TB are treated for 9–12 months, while children with TB meningitis are treated with four drugs (ethionamide replacing ethambutol) for 6 months if HIV-uninfected and 9 months if HIV-infected [17]. DR-TB regimens are individualized for children based on the drug susceptibility test (DST) results of the child’s *M.tb* isolate, or in the case of clinically diagnosed TB, of the adult source case’s isolate, with treatment ranging from 12 to 24 months.

### Study design and population

A prospective cohort study design was used to identify all children < 13 years of age (based on paediatric admission criteria at TBH) routinely diagnosed with or treated for TB at TBH during 2012.

### Surveillance strategies

Prospective hospital-based surveillance activities conducted as part of this study provided the foundation for a health system strengthening intervention for paediatric TB at TBH. In addition to active clinical and laboratory surveillance activities, support of TB referral services between hospital and community-based PHC facilities was provided. This included TB education to parents/caregivers, supporting ward personnel with the referral process, and telephonic follow-up with parents and PHC facilities following discharge to ensure continuity of care. All health system strengthening activities were implemented as part of an integrated package of TB care for children at TBH.

Clinical hospital-based surveillance: a dedicated research team including a nurse practitioner and lay healthcare worker, conducted daily clinical surveillance (Monday-Friday) in all 10 medical and surgical paediatric wards to identify children diagnosed with or treated for TB during 2012. A paper-based childhood TB tracking system (register) was implemented in all paediatric wards, outpatient services and emergency department, to serve as a communication tool between clinical and research personnel. Detailed information regarding clinical surveillance was communicated to all clinical service personnel at the start of the study, and regular feedback and training was given at paediatric departmental meetings.

Laboratory hospital-based surveillance: a dedicated laboratory-based surveillance officer identified all specimens culture-positive for *M. tb* at the microbiology laboratory at TBH. Laboratory protocols for TB culture during this time period have been described previously [10]. This study was implemented prior to the rollout of Xpert MTB/RIF (Cepheid, Sunnydale, CA) for children in this setting. Information on culture-positive specimens was communicated weekly by the laboratory to the clinical team.

### Data collection

Clinical information was captured on standard case report forms following review of clinical patient records and laboratory data. Children re-admitted during the study period were only included once. Information on TB treatment outcomes was obtained through probabilistic record linkage [18] with the 2 provincial electronic TB registers (2011–2013) using 4 variables (name, surname, gender and date of birth). All matches were manually reviewed before inclusion. If outcome information was not found in the TB registers, additional information was obtained from repeated reviews of medical records and telephonic contact with the healthcare facilities where children were discharged to (TB hospitals and PHC facilities). The National Health Laboratory Service database was also systematically surveyed for follow-up TB microbiological investigations in study participants with a culture-confirmed diagnosis. Data were dual captured in an access-controlled database and de-identified as soon as record linkages were completed.

### Definitions

Hospital visits resulting in overnight admission were classified as in-patient visits. Care pathways included both TB diagnosis pathways in relation to presentation to TBH as well as referral pathways to continue TB care on hospital discharge.

The spectrum of TB disease was classified as follows: pulmonary TB (PTB) only, EPTB only, or both PTB and EPTB. Intra-thoracic lymphadenopathy was classified as PTB. Large/loculated pleural effusions and/or miliary TB were classified as both PTB and EPTB.

We applied standard TB treatment outcome definitions as per NTP guidelines [2]: *Cured:* Children who were sputum smear-positive for acid-fast bacilli pre-treatment and who were sputum smear-negative in the last month of treatment and on at least one previous occasion, at least 30 days apart. For study purposes, we also included children with a positive culture for *M. tb* (even if smear-negative), and who had at least one follow-up negative culture before the end of treatment. *Completed treatment:* Children who had completed treatment, but did not meet the criteria for either cure or treatment failure. This category included children with bacteriological confirmation at diagnosis, but no documented follow-up bacteriological sample. Children who had documentation of having received their last month of treatment in hospital were also included, even if no further follow-up was documented. *Lost to follow-up:* Children whose treatment was interrupted for at least 2 consecutive months. For study purposes, all children who did not have follow-up information after hospital-discharge and who did not complete treatment in hospital, or where no formal outcome was assigned, were also classified as lost to follow-up. *Died*: Children who died for any reason during the course of TB treatment. For study purposes we also included in-hospital deaths prior to initiation of treatment (e.g. if TB was culture-confirmed only after the child died). *Treatment failure*: Children who remained bacteriologically positive at 5 months or later after starting treatment. *Transferred out:* Children who were transferred to another district and for whom the treatment outcome was not known. Favourable treatment outcomes were combined as cured or treatment completed. Unfavourable treatment outcomes included lost to follow-up (including not evaluated), died, treatment failure, and transferred out.

### Statistical analysis

Given the long-standing history of hospital-based laboratory surveillance at TBH, the primary aim was to characterise the cohort of children with a clinical/presumed diagnosis of TB, who would not be identified by existing microbiological laboratory surveillance. Children were therefore grouped into those identified through laboratory surveillance and those identified through clinical surveillance only, acknowledging that a proportion of children identified through laboratory surveillance would have also been identified through clinical surveillance.

Descriptive and summary statistics were used to calculate numbers and percentages of the overall disease burden, spectrum, clinical characteristics, referral pathways and TB treatment outcomes. The following variables were included in analysis: demographics (age and sex), TB treatment and exposure history, HIV status and related variables, nutritional status, spectrum and type of TB disease, referral care pathways, and TB treatment outcomes. Odds ratios (ORs) and 95% confidence intervals (CIs) were calculated in univariate analysis to investigate and quantify differences between children identified through the two hospital-based surveillance strategies explained above (laboratory vs clinical only). Weights were transformed to z-scores using reference data available from the 1990 British Growth Reference [19]. Statistical analysis was completed using STATA SE version 14 software (StataCorp LP, Texas, USA).

### Ethical considerations

Ethics approval was obtained from Stellenbosch University Health Research Ethics Committee (N11/09/28) and provincial and municipal authorities. As the study was implemented as part of standard clinical care, a waiver of individual informed consent was granted. The STROBE guidelines for reporting of cohort studies were followed [20].

## Results

**Fig. 1:**
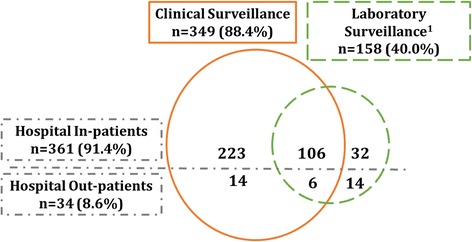
Overview of the yield of complementary hospital-based surveillance strategies to identify children with tuberculosis. ^1^Of the 46 children identified only through laboratory surveillance, 27 children were started on TB treatment prior to TBH discharge but missed by clinical hospital surveillance; 19 were only started on TB treatment at a follow-up visit

During 2012, 395 children (< 13 years of age) were managed for TB at TBH. Figure 1 provides an overview of the yield of clinical and laboratory hospital-based surveillance strategies. Although the majority of children (349, 88.4%) were identified through clinical surveillance, laboratory-based surveillance identified 158 (40.0%) children with culture-confirmed TB. Laboratory surveillance identified 46 (11.6%) children who were not detected by clinical ward-based surveillance. Clinical surveillance identified 237 (60%) of children that would have been missed by existing laboratory hospital-based surveillance.

Demographic and clinical characteristics for all children are presented in Table 1. The median age was 2.4 years (interquartile range [IQR] 1.0–5.1) with 180 (45.6%) children less than 2 years of age; 213 (53.9%) were male. Hospital admission was required for 361 (91.4%) children, of whom 238 (65.9%) was admitted for > 1 week. Twenty-six (7%) children had a history of previous TB treatment. A history of TB exposure was reported in 213 (55.5%), of which 143 (73.3%) occurred in the household. HIV status was documented in 370 (93.7%), with 90/370 (24.3%) HIV-infected and 24/90 (26.7%) diagnosed with HIV during hospital admission. Of the 66 children known to be HIV-infected at the time of admission, 44 (66.7%) were already on antiretroviral therapy (ART). By 2 weeks following TBH discharge, 83 (92.2%) were receiving ART. Of all TB cases, 113 (29.5%) children had a weight-for-age Z-score < − 3. PTB only was diagnosed in 207/395 (52.4%), both PTB and EPTB in 117 (29.6%) and the remaining 71 (18.0%) children had only EPTB. TB meningitis and miliary TB were diagnosed in 62 (15.7%) and 24 (6.1%) children, respectively; 9 children had both. Of 393 children started on treatment, 342 (87.0%) were treated for DS-TB, 9 (2.3%) for isoniazid mono-resistant TB, 4 (1.0%) for rifampicin mono-resistant TB, 34 (8.7%) for multidrug-resistant TB, 3 (0.8%) for pre-extensively drug-resistant TB and 1 (0.2%) for extensively drug-resistant TB. Nine (2.3%) children died during hospital admission; 2 with culture-confirmed DS-TB (results available only after death) died before TB treatment was initiated.

**Table 1:** Demographic and clinical characteristics of children with tuberculosis managed at Tygerberg Hospital during 2012

	Number (%)^a^ *n* = 395
Demographics and characteristics at hospital admission	
Age (years)	
0 - < 2	180 (45.6)
2 - < 5	116 (29.4)
5 – < 13	99 (25.1)
Male sex	213 (53.9)
In-patient admissions	361 (91.4)
Duration of hospitalisation for in-patients (*n* = 361)	
≤ 1 week	123 (34.1)
2–3 weeks	168 (46.5)
≥ 4 weeks	70 (19.4)
TB history	
Previous TB treatment reported	26/371 (7.0)
Any TB exposure reported	213/384 (55.5)
Household TB exposure reported (level of TB exposure documented; *n* = 195)	143/195 (73.3)
HIV and nutritional status	
HIV status documented	370 (93.7)
HIV-infected	90/370 (24.3)
Diagnosed with HIV before hospital admission	66/90 (73.3)
On ART at hospital admission	44/66 (66.7)
Median CD4 percentage^b^ (inter-quartile range)	17.0 (11.6–23.0)
Median CD4 absolute value^b^ (inter-quartile range)	593 (274–1,150)
On ART within 2 weeks after hospital discharge	83/90 (92.2)
Weight-for-Age Z-score < −3^c^	113/383 (29.5)
TB disease characteristics	
Bacteriologically confirmed TB (culture-positive for *M. tb*)	158 (40.0)
Spectrum of disease	
Pulmonary TB (PTB) only	207 (52.4)
Both PTB and extra-pulmonary TB (EPTB)	117 (29.6)
EPTB only	71 (18.0)
Disseminated TB (TB Meningitis and Miliary TB)	77 (19.5)
Spectrum of EPTB	
TB Meningitis^d^	62 (15.7)
Miliary TB^d^	24 (6.1)
TB pleural effusion/empyema^e^	35 (8.9)
Abdominal TB only	16 (4.1)
Central Nervous System TB (not TB meningitis)	8 (2.0)
Musculoskeletal TB	19 (4.8)
Pericardial effusion	3 (0.8)
Cutaneous TB	1 (0.3)
Renal TB	1 (0.3)
Peripheral lymphadenitis	28 (7.1)
Type of TB treatment^f^	
First-line regimen (drug-susceptible TB) ^f^	342/393 (87.0)
INH mono-resistant treatment regimen	9/393 (2.3)
Rif mono-resistant treatment regimen	4/393 (1.0)
MDR treatment regimen	34/393 (8.7)
Pre-XDR treatment regimen	3/393 (0.8)
XDR treatment regimen	1/393 (0.2)
Deaths during hospital admission	9 (2.3)

^a^The denominator was 395, unless otherwise specified due to missing data

^b^Median value of available CD4 laboratory result within 2 weeks before and after hospital admission (not available for 32/90 HIV-infected children)

^c^Weights were transformed to z-scores using the reference data available from the 1990 British Growth Reference

^d^Includes 9 children that had both miliary TB and TB meningitis

^e^TB pleural effusion/empyema includes 4 children with abdominal TB as well

^f^Excludes two children who died in hospital before any treatment was started, but subsequently had a drug-susceptible mycobacterial culture

*TB* Tuberculosis, *HIV* Human immune-deficiency virus, *ART* Antiretroviral treatment, *M. tb* = *Mycobacterium tuberculosis*, *INH* Isoniazid, *Rif* Rifampicin, *MDR* Multidrug-resistant, *XDR* Extensively drug-resistant

Ninety-one (23.0%) children had been diagnosed with TB prior to admission at TBH (Table 2); of these, 76 (83.5%) were diagnosed with TB at hospital level and 41 (45.1%) were diagnosed less than 2 weeks before admission to TBH. At the time of discharge (*n* = 386 children), multiple referral pathways to continue TB care were followed: 244 (63.2%) were discharged to community-based PHC facilities, 82 (21.2%) were transferred to dedicated TB hospitals, 28 (7.3%) were discharged home with monthly outpatient follow-up visits at TBH, 23 (6.0%) were down-referred to secondary hospitals, 8 (2.1%) were referred to medium-term care facilities and 1 (0.3%) child completed TB treatment during a non-TB related admission at TBH.

**Table 2:** Care pathways and treatment outcomes^a^ of children with tuberculosis managed at Tygerberg Hospital during 2012

TB diagnosis pathways in relation to Tygerberg Hospital (TBH) presentation	*n* = 395 (%)
TB diagnosis made during/following current presentation to TBH	304 (77.0)
TB diagnosis made prior to TBH admission	91 (23.0)
Duration of TB treatment at time of TBH admission (*n* = 91)	
0–14 days on TB treatment	41 (45.1)
15–60 days on TB treatment	23 (25.3)
> 60 days on TB treatment	27 (29.7)
Level of care at which TB diagnosis was made (*n* = 91)	
Diagnosed at hospital level	76 (83.5)
Diagnosed at a community primary health care (PHC) facility	15 (16.5)
Discharge referral pathways to continue TB care^b^	
Community-based TB services (PHC facilities)	244/386 (63.2)
Hospital-based outpatient follow–up at TBH	28/386 (7.3)
TB hospitals	82/386 (21.2)
Other^c^	32/386 (8.3)
TB treatment outcomes for children treated as drug-susceptible TB^d^	*n* = 344 (%)
* Favourable treatment outcomes (Total)*	*300 (87.2)*
Cured	12 (3.5)
Treatment completed	288 (83.7)
* Unfavourable treatment outcomes (Total)*	*44 (12.8)*
Died^d^	17 (4.9)
Lost to-follow up^e^	23 (6.7)
Treatment failure	1 (0.3)
Transferred out	3 (0.9)
TB treatment outcomes for children treated as drug-resistant TB^f^	*n* = 51 (%)
* Favourable treatment outcomes (Total)*	*50 (98.0)*
Cured	14 (27.5)
Treatment completed	36 (70.6)
* Unfavourable treatment outcomes (Total)*	*1 (2.0)*
Died	0
Lost to-follow up	1 (2.0)
Treatment failure	0
Transferred out	0

^a^Outcome information was firstly collected through probabilistic record linkage with electronic TB treatment registers. If information was not found in the registers, additional follow up information on outcomes were obtained from repeated reviews of medical records, telephonic contact with the facilities patients were discharged to (TB hospitals and community PHC facilities), as well as the National Health Laboratory Service database

^b^Excludes children who died during hospital admission (*n* = 9)

^c^Includes referrals to secondary hospitals (*n* = 23), chronic medium term care facilities (*n* = 8) and one child that completed TB treatment during a non-TB related admission

^d^Includes two children that died during hospital admission before TB treatment was initiated, but subsequently had a positive culture for drug-susceptible TB

^e^Includes 10 children (2.9%) for whom no follow up documentation could be found in any available data sources

^f^Includes 9 children with isoniazid mono-resistant TB, 4 with rifampicin mono-resistant TB, 34 with multidrug-resistant TB, and 4 with extensively drug-resistant TB

*TB* Tuberculosis, *TBH* Tygerberg Hospital, *PHC* Primary healthcare

Final TB treatment outcomes overall were excellent, with favourable treatment outcomes in 300/344 (87.2%) children treated for DS-TB, and 50/51 (98%) treated for DR-TB (Table 2). Despite the overall favourable outcomes, mortality was substantial amongst children diagnosed with DS-TB (17; 4.9%).

Results of the analysis comparing characteristics between children identified through the two hospital-based surveillance strategies (laboratory culture-confirmed vs. clinical diagnosis only), are presented in Table 3. Laboratory surveillance was more likely to identify older children (5–<13 years of age) compared to children < 2 years of age (OR 1.7; 95% CI 1.0–2.8; *p* = 0.042), children with EPTB (OR 2.3; 95% CI 1.5–3.6; *p* < 0.001) especially in the presence of miliary TB (OR 6.3; 95% CI 2.3–17.8; *p* < 0.001), and children who died during hospital admission (OR 5.4; 95% CI 1.1–26.9; *p* = 0.033). TB/HIV co-infected patients (OR 0.3; 95% CI 0.2–0.5; *p* < 0.001) and in-patients (OR 0.4; 95% CI 0.2–0.9; *p* = 0.019) were less likely to be detected by laboratory surveillance. No significant differences were observed for sex, duration of hospital admission, TB history, documentation of HIV status, weight-for-age Z-score < − 3, presence of TB meningitis, type of TB treatment, discharge referral pathways and TB treatment outcomes.

**Table 3:** Comparing clinical characteristics, referral pathways and TB treatment outcomes of children by two complementary hospital-based surveillance strategies

	All identified through laboratory surveillance *n* = 158 (40.0%)^a^	Identified through clinical surveillance only *n* = 237 (60.0%)^a^	Odds Ratio (95% CI)	*p*-value
Demographics and admission characteristics				
Age (years)				
0 - < 2	63 (39.9)	117 (49.4)	Reference	
2 – < 5	48 (30.4)	68 (28.7)	1.3 (0.8–2.1)	0.269
5 – < 13	47 (29.8)	52 (21.9)	1.7 (1.0–2.8)	0.042
Male sex	77 (48.7)	136 (57.4)	0.7 (0.5–1.1)	0.092
In-patient admissions	138 (87.3)	223 (94.1)	0.4 (0.2–0.9)	0.019
Duration of hospitalisation				
≤ 1 week	46 (33.3)	77 (34.5)	Reference	–
2–3 weeks	68 (49.3)	100 (44.8)	1.1 (0.7–1.8)	0.595
≥ 4 weeks	24 (17.4)	46 (20.6)	0.9 (0.5–1.6)	0.666
TB History				
Previous TB treatment reported	7/150 (4.7)	19/221 (8.6)	0.5 (0.2–1.3)	0.146
Any TB exposure reported	77/154 (50.0)	136/230 (59.1)	0.7 (0.5–1.0)	0.078
Household TB exposure reported	50/69 (72.5)	93/126 (73.8)	0.9 (0.5–1.8)	0.781
HIV and nutritional status				
HIV status documented	146 (92.4)	224 (94.5)	0.7 (0.3–1.6)	0.400
HIV-infected (of those tested)	18/146 (12.3)	72/224 (32.1)	0.3 (0.2–0.5)	< 0.001
Weight-for-Age Z-score < − 3^b^	53/153 (34.6)	60/230 (26.1)	1.5 (1.0–2.3)	0.073
TB disease characteristics				
Spectrum of TB disease				
PTB only	63 (39.9)	144 (60.8)	Reference	
ETPB with/without PTB	95 (60.1)	93 (39.2)	2.3 (1.5–3.6)	< 0.001
Disseminated TB				
TB Meningitis^c^	20 (12.7)	42 (17.7)	0.7 (0.4–1.2)	0.176
Miliary TB^c^	19 (12.0)	5 (2.1)	6.3 (2.3–17.8)	< 0.001
Type of TB treatment				
First-line regimen	134/156 (85.9) ^d^	208/237 (87.8)	Reference	–
Any drug-resistant regimen	22/156 (14.1) ^d^	29/237 (12.2)	1.2 (0.7–2.1)	0.591
Deaths during hospital admission	7 (4.4)	2 (0.8)	5.4 (1.1–26.9)	0.033
Discharge referral pathway for continuation of TB care^e^				
Community-based PHC facilities	93/151 (61.6)	151/235 (64.3)	Reference	
Hospital-based outpatient follow-up	9/151 (6.0)	19/235 (8.1)	0.8 (0.3–1.8)	0.537
TB hospitals	37/151 (24.5)	45/235 (19.2)	1.3 (0.8–2.2)	0.263
Other^f^	12/151 (8.0)	20/235 (8.5)	1.0 (0.5–2.1)	0.946
TB treatment outcomes (DS-TB)				
Favourable	119/136 (87.5)	181/208 (87.0)	1.0 (0.5–2.0)	0.896
Unfavourable	17/136 (12.5)	27/208 (13.0)	Reference	
TB treatment outcomes (DR-TB)				
Favourable	22/22 (100)	28/29 (96.6)	–	1.000
Unfavourable	0/22 (0.0)	1/29 (3.5)		

^a^The denominator was 158 or 237 respectively, unless otherwise specified due to missing data

^b^Weights were transformed to z-scores using the LMS method and the reference data available from 1990 British Growth Reference^.^; missing admission weights (*n* = 10)

^c^Inlcudes 9 children that had both miliary TB and TB meningitis

^d^Excludes two children that died during hospital admission before TB treatment was initiated, but subsequently had a positive culture for drug-susceptible TB

^e^Excludes children who died in-hospital (*n* = 9)

^f^Includes referrals to secondary hospitals (*n* = 23), chronic medium term care facilities (*n* = 8) and one child that completed TB treatment during a non-TB related admission

*TB* Tuberculosis, *CI* Confidence interval, *HIV* Human immune-deficiency virus, *ART* Antiretroviral treatment, *PHC* Primary healthcare, *DS* Drug-susceptible, *DR* Drug-resistant

## Discussion

In this TB-endemic setting, our study identified a very large burden of childhood TB managed at referral hospital level, with almost 400 children during a one-year period at a single hospital. Clinical surveillance identified 237 (60%) children in addition to the existing laboratory surveillance. Despite the majority of children being young (74.9% < 5 years of age), the diagnosis was bacteriologically (culture) confirmed in 40% of children. Such a high proportion of confirmed diagnoses is probably the result of appropriate specimen collection (standard of care is at least 2 respiratory specimens in children < 5 years of age or other indicated specimens for EPTB) and the high proportion of severe forms of TB, which is associated with higher yield by culture [11]. Nearly 20% of children had disseminated TB (TB meningitis or miliary TB), associated with high morbidity and mortality [21–23]. Almost a quarter of children were already on TB treatment at the time of admission, and only 63% of children were referred to community-based PHC facilities on discharge. This reflects both the complexity of TB disease in children with TB managed at referral hospital level, as well as the as-yet under-appreciated movement of children between different levels of healthcare services during the course of their TB diagnosis and treatment. The high proportion of children with drug-resistant TB (12.9%) reflects that TBH is a provincial centre of expertise for the management of DR-TB in children with a dedicated paediatric DR-TB out-patient service and clinical experts.

To our knowledge, this is the first study to characterize the TB disease burden, spectrum, clinical care pathways and final TB treatment outcomes of childhood TB (including both confirmed and clinically diagnosed cases) managed within routine health care services at a large referral hospital in South Africa. This study therefore comprehensively captured the true burden and spectrum of paediatric TB in a large hospital in a high TB burden setting. We also identified important clinical and care pathway differences between children identified through existing laboratory surveillance at this hospital and those with a presumed diagnoses identified through additional clinical surveillance.

There are some studies reporting on childhood TB at tertiary/referral hospitals in Africa. An Ethiopian study reported 491 children treated from 2009 to 2014, and also found a high proportion (49.4%) of EPTB [24]. However, children in that study were considerably older, with only 107 (21.8%) children < 5 years of age. In the Ethiopian study, 82 (28%) children were HIV-infected, but the HIV status was unknown in a large proportion (41%) [24]. Another study from Kinshasa, Democratic Republic of Congo, reported similar proportions of children with EPTB (159/283; 56.1%) and 32/97 (33%) were culture-positive for *M. tb* during 2005–2011 [25]. However, cultures were only performed on a third of children, and similar to the Ethiopian study, the age distribution of children was older with only 87 (30.7%) less than 7 years of age. In this study only 2.5% of children were HIV-infected, but in 75% the status was not known [25]. The burden of childhood TB reported at these hospitals was considerably lower than what we have observed in our study (491/5 years; 283/7 years; 395/1 year).

Younger and HIV-infected children were less likely to be identified by existing laboratory surveillance, i.e. they were less likely to have culture-confirmed TB. This may reflect clinicians’ lower threshold to diagnose TB in children at high risk of developing more severe forms of TB. Older children, and those with EPTB and disseminated disease were more likely to be bacteriologically confirmed, possibly reflecting a higher likelihood to develop adult-type pulmonary disease, with a higher bacillary burden and the ability to produce and expectorate sputum [3]. Laboratory surveillance also identified a large proportion of children with EPTB where pathological specimens are more readily obtained (e.g. peripheral lymphadenitis). However, laboratory surveillance in isolation is likely to still miss certain types of EPTB, especially TB meningitis (extremely paucibacillary in the absence of additional PTB), TB in HIV co-infected children and in very young children.

Multiple studies have shown that adult and paediatric TB patients are at risk of loss to follow-up when moving between different levels of health care, especially if they access care at large hospitals [5, 26, 27]. Our study found considerable movement of children between community PHC facilities, general hospitals and TB hospitals during their TB episode, possibly increasing the risk of unfavourable outcomes and incomplete NTP surveillance data. Almost half of the children received in-patient tertiary care for 2–3 weeks, and a fifth of the children were referred to TB hospitals for specialized care after discharge. Only approximately two-thirds were referred for community TB care. An additional advantage of hospital-based surveillance for paediatric TB, is to provide information on hospital admissions and to inform resource allocation to improve management of children with TB. Despite the relative resource intensity of clinical surveillance in a large hospital, a major advantage is the ability to complete linkage to care and real-time follow-up, which is not feasible when using laboratory-based surveillance only due to long turn-around time in culture results. This will be only partially addressed by the shorter turn-around time of new microbiological methods such as Xpert MTB/RIF, as current molecular diagnostic methods are less sensitive than culture in paucibacillary TB [28].

Overall TB treatment outcomes were very good despite the young age and the high proportion of children with severe TB and co-morbidities (HIV co-infection and malnutrition), especially in children with DR-TB. The higher proportion of favourable outcomes amongst children with DR-TB is likely partly a function of the established high-quality clinical program for the management of children with DR-TB in this setting and the more complete follow-up data available in this group of children. The treatment outcomes observed in the drug-resistant group are similar to those described previously in this setting (92% favourable treatment outcomes; *n* = 149) [29]. We report favourable outcomes similar to those observed in a study evaluating routine community-based surveillance data for children treated for DS-TB in this setting (85.9%), where children typically would have limited/uncomplicated disease [30]. However, mortality among children with DS-TB (4.9%) was considerably higher than reported from routine community-based NTP surveillance data (0.7%) from the same setting. Laboratory hospital-based surveillance identified the majority of children who died from TB during hospitalization (two never started TB treatment), and can potentially provide important information on paediatric mortality. This highlights the importance of hospital-based surveillance strategies to better capture TB mortality in children. However, TB treatment outcome data do not provide information on morbidity, hospital admission requirements, healthcare costs and the lifelong disabilities suffered by children and families resulting from severe forms of TB like TB meningitis, osteoarticular disease (especially spinal TB), and chronic lung disease from PTB. A long-term outcome study evaluating children with TB meningitis found that only 1 in 5 children functioned normally at long-term follow-up (median follow-up time after completion of anti-tuberculosis therapy: 6 years 6 months), with 80% suffering cognitive impairment, highlighting the extreme morbidity and life-long implications of TB meningitis [31]. Hospital-based surveillance data can therefore provide important information to the NTP to guide appropriate planning and resource allocation.

Our study had several limitations. All children diagnosed with or managed for TB by routine healthcare services clinicians were included. Over-diagnosis in the clinically diagnosed group was therefore possible, especially among very young and HIV-infected children. However, given the high proportion of children with a confirmed diagnosis in this cohort, and the specialised nature of clinical services at the tertiary referral hospital, we expect the proportion of children who did not truly have TB to be low. Operational implementation of ongoing clinical surveillance in a hospital with 10 paediatric wards, several outpatients and an emergency unit, staffed with more than a 100 clinicians was challenging. Study personnel had to rely on hospital clinicians to record all children diagnosed over weekends and evaluated as out-patients in paper-based registers, and clinical surveillance may have missed some patients. The documented burden of disease in our study would therefore represent the minimum burden of paediatric TB managed at TBH.

## Conclusion

Complementary hospital-based surveillance strategies are essential to provide a comprehensive picture of the burden, spectrum, referral pathways and outcomes of children with TB managed at referral hospital level. It is important to understand setting-specific and epidemiological differences when interpreting NTP TB data from different sources of surveillance. Integration of electronic patient management systems, including hospital data, could simplify and improve the accuracy of TB reporting in future. In settings where hospitals do not function as TB reporting units, the inclusion of hospital surveillance data within NTP surveillance data should be prioritised.

## Abbreviations

ART: Antiretroviral therapy
CI: Confidence interval
DR-TB: Drug resistant TB
DS-TB: Drug susceptible TB
EPTB: Extra-pulmonary TB
*M.tb*: *Mycobacterium tuberculosis*
NTP: National Tuberculosis Program
OR: Odds ratio
PHC: Primary healthcare
PTB: Pulmonary TB
TB: Tuberculosis
TBH: Tygerberg Hospital
WHO: World Health Organisation
